# ﻿*Pileadanxiaensis* (Urticaceae), a new species in the Danxia landform from Guangdong, China including a description of the entire chloroplast genome

**DOI:** 10.3897/phytokeys.204.86857

**Published:** 2022-08-19

**Authors:** Long-Fei Fu, Chi Xiong, Alexandre K. Monro, Qiang Fan, Zai-Xiong Chen, Fang Wen, Zi-Bing Xin, Yi-Gang Wei, Wen-Bo Liao

**Affiliations:** 1 Guangxi Key Laboratory of Plant Conservation and Restoration Ecology in Karst Terrain, Guangxi Institute of Botany, Guangxi Zhuang Autonomous Region and Chinese Academy of Sciences, CN-541006 Guilin, China; 2 The Royal Botanic Gardens, Kew, Identification & Naming Department, Richmond, Surrey TW9 3AE, UK; 3 State Key Laboratory of Biocontrol, School of Life Sciences, Sun Yat-sen University, Guangzhou 510275, China; 4 Guangdong Provincial Key Laboratory of Plant Resources, School of Life Sciences, Sun Yat-sen University, Guangzhou 510275, China; 5 Administrative Commission of Danxiashan National Park, Shaoguan 512300,China

**Keywords:** Danxia landscape, new taxon, plastome, taxonomy

## Abstract

*Pileadanxiaensis* L.F.Fu, A.K.Monro & Y.G.Wei, a new species of Urticaceae from Danxia landform, Guangdong, China, is described and photographed. Phylogenetic analyses based on three DNA regions (ITS, *trnL-F* and *rbcL*) suggest that the new species belongs to P.sect.Pilea. Within the section, the new species is morphologically most similar to *P.sinocrassifolia* and *P.peploides*. Plastid genome and ribosomal DNA (rDNA) sequences of the new species are assembled and annotated. The plastid genome is 151,857 bp in length and comprises two inverted repeats (IRs) of 25,307 bp separated by a large single-copy of 82,836 bp and a small single-copy of 18,407 bp. A total of 113 functional genes are recovered, comprising 79 protein-coding genes, 30 tRNA genes, and four rRNA genes. A global conservation assessment suggests that *P.danxiaensis* should be classified as of Least Concern (LC).

## ﻿Introduction

*Pilea* Lindl. is the largest genus in the Urticaceae that comprises *ca* 715 species worldwide (Monro 2004; Fu et al. 2022). *Pilea* has a pantropical and subtropical distribution with the exception of Australia and New Zealand and is characterized by succulent herbs, shrubs and epiphytes and many point-endemic species. Outside the Neotropics, Indomalaya is the main center of diversification for *Pilea* (Monro 2006; Fu et al. 2022). Within Indomalaya, China contains more than 90 species (Chen and Monro 2003, Chen et al. 2007; Monro et al. 2012; Wang 2014, 2016; Fu et al. 2017; Yang et al. 2018).

A recent systematic study has demonstrated that *Pilea* is monophyletic after the exclusion of species of *Achudemia* and *Lecanthus* (Fu et al. 2022). The newly circumscribed genus has been classified into eight sections: Pileasect.Tetraphyllae Y.G.Wei & A.K.Monro, sect. Trimeris Y.G.Wei & A.K.Monro, sect. Lecanthoides C.J.Chen, sect. Angulatae L.F.Fu & Y.G.Wei, sect. Tetrameris C.J.Chen, sect. Verrucosae L.F.Fu & Y.G.Wei, sect. Plataniflorae L.F.Fu & Y.G.Wei and sect. Pilea (Fu et al. 2022). These sections can be delimited by leaf margin morphology, stipule length, inflorescence architecture, flower sepal number and achene ornamentation (Fu et al. 2022).

While conducting field investigations into the Danxia flora of Guangdong, China, we encountered an unknown species of *Pilea* with 3-parted female flowers, 4-parted male flowers, short stipules (≤10 mm), entire leaf margins and ornamented or rarely smooth achenes that placed it within Pileasect.Plataniflorae or sect. Pilea (Fu et al. 2022). After a thorough literature survey and review of herbarium specimens at IBK, IBSC, K, PE and SYS, along with molecular studies, we confirmed that this plant was a hitherto undescribed species of Pileasect.Pilea.

## ﻿Materials and methods

### ﻿Morphological observations and conservation assessment

All morphological characters were observed from field and herbarium specimens using an Olympus SZX16 binocular microscope (Japan). For achene morphology, we also undertook scanning electron micrograph (SEM) observations. Achene materials were collected from specimens, dried, and mounted using double-sided adhesive tape and coated with gold using a sputter coater. The materials were then observed and photographed under a ZEISS EVO18 scanning electron microscope. At least ten achenes were used to determine their size and surface ornamentation.

A species conservation assessment was undertaken for the new species described here using IUCN criteria (IUCN 2019). Calculations of the extent of occurrence (EOO) and area of occupation (AOO) were undertaken using the online conservation assessment tool GeoCATAT (Bachman et al. 2011). The AOO was calculated using a cell width of 2 km as recommended by IUCN (2019).

### ﻿Genomic DNA extraction and sequencing

Leaf material for DNA extraction was dried using silica gel (Chase and Hills 1991). Genomic DNA was extracted using a modified CTAB protocol (Chen et al. 2014). The total gDNA sample was sent to Majorbio (http://www.majorbio.com/, China) for library construction and next-generation sequencing. Short-insert (350 bp) paired-end read libraries preparation and 2 × 150 bp sequencing were performed on an Illumina (HiSeq4000) genome analyzer platform. Approximately 2 Gb of raw data for the new species was filtered using the FASTX-Toolkit to obtain high-quality clean data by removing adaptors and low-quality reads (http://hannonlab.cshl.edu/fastx_toolkit/download.html).

### ﻿Plastid genome and ribosomal DNA (rDNA) assembly and annotation

Clean reads were paired and imported in Geneious Prime (Kearse et al. 2012). For plastid genome assembly, the clean reads were mapped to published plastid genome sequence as reference (Fu et al. 2019) using the Fine Tuning option in Geneious Prime (iterating set as 10 times) to exclude nuclear and mitochondrial reads. Then, de novo assembly was performed using Geneious Prime with a medium-low sensitivity setting to assemble plastid genome sequence. The generated contigs were mapped by the clean reads using the Fine Tuning option in Geneious Prime (iterating set as 10 times) to fill gaps. Contigs were able to be concatenated using the Repeat Finder option implemented in Geneious Prime until a ~130 kb contig (including SSC, IR and LSC) being built. The Inverted repeat (IR) region was determined by the Repeat Finder option in Geneious Prime and was reverse copied to obtain the complete plastid genome. The annotation approach of the assembled plastid genome was performed using CPGAVAS2 and PGA (Qu et al. 2019; Shi et al. 2019). The process of rDNA assembly is similar to the above with the exception of different reference (Fu et al. 2021) and iterating as none. The annotation approach of rDNA was performed using Annotate option in Geneious Prime.

### ﻿Phylogenetic analyses

We generated a phylogeny using sequences data from previous phylogenies of *Pilea* s.l. (Fu et al. 2022). We extracted three DNA regions (ITS, *trnL-F* and *rbcL*) from assembled rDNA and complete plastid genome sequences, respectively, of the new species and downloaded all sequences data used in Fu et al. (2022) from GenBank (details see Suppl. material 1). This resulted in 145 accessions representing 131 taxa in total, 112 taxa of which belong to *Pilea* s.l. as in-group, and 21 species of which belong to *Elatostema* s.l., other tribes of Urticaceae, Moraceae and Cannabaceae, as out-group (Appendix 1). Three datasets (ITS, *trnL-F* and *rbcL*) were aligned independently using multiple alignment using fast Fourier transform (MAFFT) version 7.0 (Katoh and Standley 2013) with default settings, followed by manual adjustment. As there was no incongruence that affected the topology of the ingroup taxa as described in Fu et al. (2022), phylogenies were reconstructed based on the combined dataset using Maximum Likelihood (ML) and Bayesian inference (BI). The BI and ML analyses were performed following Fu et al. (2022).

## ﻿Results

### ﻿Characteristics of the complete plastid genome and ribosomal DNA

**Figure 1.**
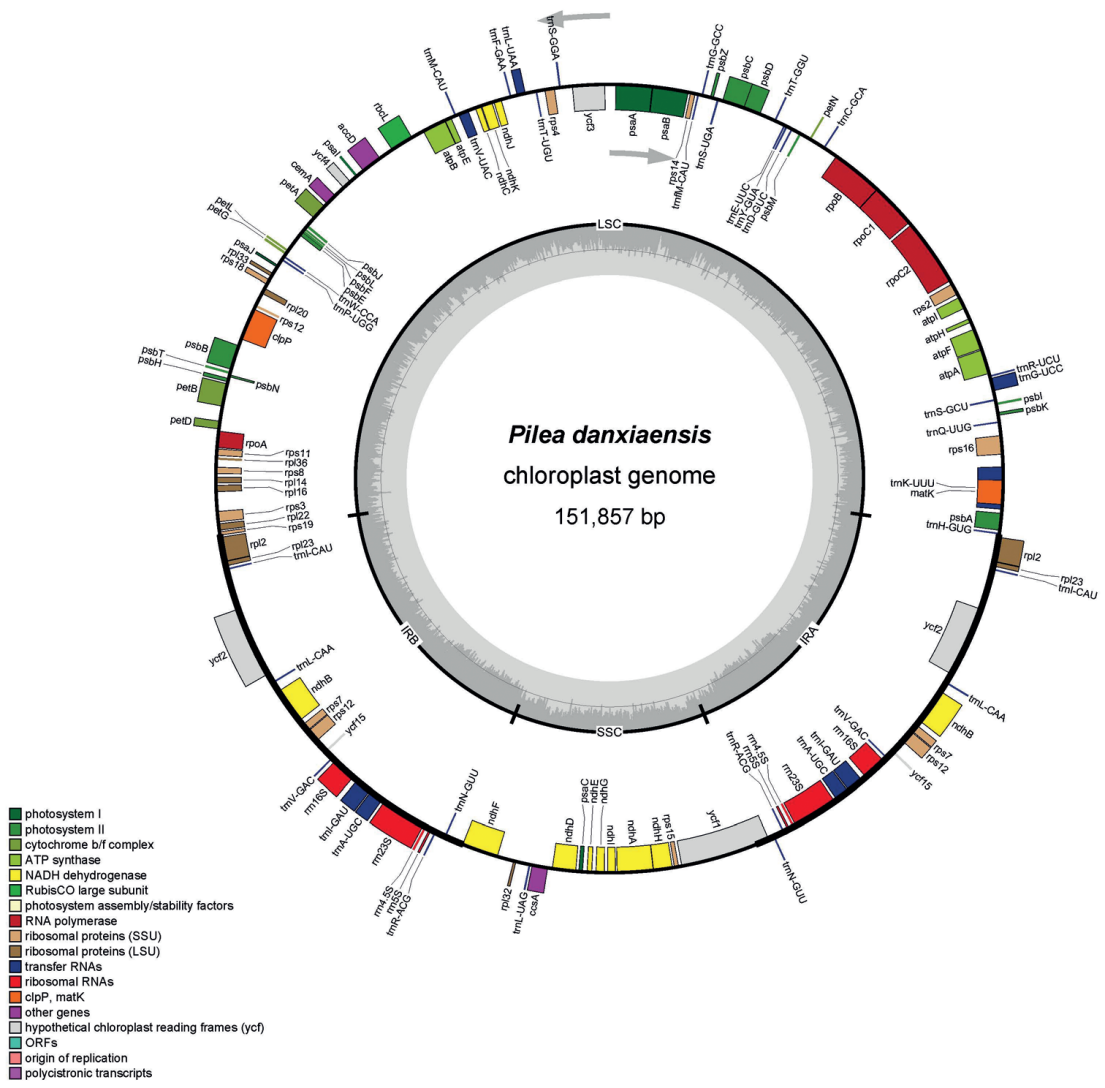
Plastid genome map of *Pileadanxiaensis*. The thick lines on the outer complete circle identify the inverted repeat regions (IRa and IRb). The innermost track of the plastome shows the GC content. Genes on the outside and inside of the map are transcribed in clockwise and counter directions, respectively.

The complete plastid genome and ribosomal DNA (rDNA) sequences of *Pileadanxiaensis* comprised 151,857 bp (Fig. 1) and 5,788 bp, respectively. The characteristics and statistics of plastid genome are summarized in Tables 1 and 2.

**Table 1.** Summary of whole plastid genome of *Pileadanxiaensis*.

Characteristic	*Pileadanxiaensis*
Size (bp)	151,857
LSC length (bp)	82,836
SSC length (bp)	18,407
IR length (bp)	25,307
Number of genes	113
Protein-coding genes	79
rRNA genes	4
tRNA genes	30
LSC GC%	34.30%
SSC GC%	30.70%
IR GC%	42.80%

**Table 2.** Genes encoded in plastid genome of *Pileadanxiaensis*.

Group of genes	Gene name
tRNA genes	*trnA-UGC (×2), trnC-GCA, trnD-GUC, trnE-UUC, trnF-GAA, trnfM-CAU, trnG-GCC, trnG-UCC, trnH-GUG, trnI-CAU (×2), trnI-GAU (×2), trnK-UUU, trnL-CAA (×2), trnL-UAA, trnL-UAG, trnM-CAU, trnN-GUU (×2), trnP-UGG, trnQ-UUG, trnR-ACG (×2), trnR-UCU, trnS-GCU, trnS-GGA, trnS-UGA, trnT-GGU, trnT-UGU, trnV-GAC (×2), trnV-UAC, trnW-CCA, trnY-GUA*
rRNA genes	*rrn16 (×2), rrn23 (×2), rrn4.5 (×2), rrn5 (×2)*
Ribosomal small subunit	rps16*, rps2, rps14, rps4, rps18, rps12** (×2), rps11, rps8, rps3, rps19, rps7 (×2), rps15
Ribosomal large subunit	pl33, rpl20, rpl36, rpl14, rpl16*, rpl22, rpl2* (×2), rpl23 (×2), rpl32
DNA-dependent RNA poly merase	poC2, rpoC1*, rpoB, rpoA
Photosystem I	*psaB, psaA, psaI, psaJ, psaC*
Photosystem II	*psbA, psbK, psbI, psbM, psbC, psbZ, psbG, psbJ, psbL, psbF, psbE, psbB, psbT, psbN, psbH*
Large subunit of rubisco	*rbcL*
NADH dehydrogenase	ndhJ, ndhK, ndhC, ndhB* (×2), ndhF, ndhD, ndhE, ndhG, ndhI, ndhA*, ndhH
Cytochrome b/f complex	petN, petA, petL, petG, petB*, petD*
ATP synthase	atpA, atpF*, atpH, atpI, atpE, atpB
Maturase	*matK* (The *matK* is localized between the exons coding for the *trnK-UUU*)
Subunit of acetyl-CoA carboxylase	*accD*
Envelope membrane protein	*cemA*
Protease	clpP**
Translational initiation factor	*infA*
C-type cytochrome synthesis	*ccsA*
Conserved open reading frames	ycf3**, ycf4, ycf2 (×2), ycf1, ycf15 (×2)

Note: Genes with one or two introns are indicated by one (*) or two asterisks (**), respectively. Genes in the IR regions are followed by the (× 2) symbol.

### ﻿Phylogenetic reconstruction

**Figure 2.**
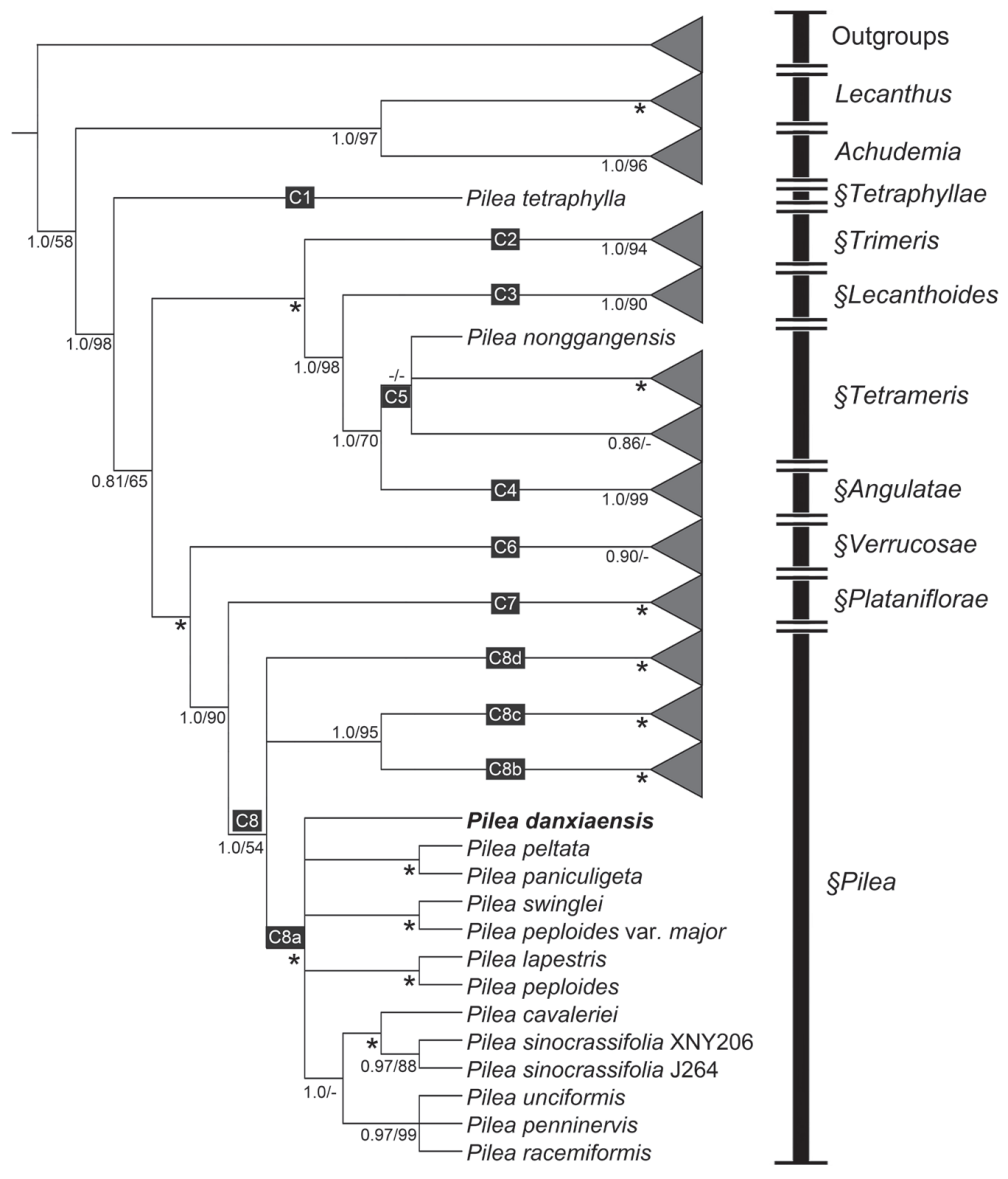
Phylogenetic tree of *Pilea* s.l. generated from Bayesian Inference (BI) of combined dataset (ITS, *trnL-trnF* and *rbcL*). Numbers below the branches indicate the posterior probability (≥0.5) of BI and bootstrap values (≥50%) of the ML analyses. ‘*’ indicates supports of 1.0/100. The bold (***Pileadanxiaensis***) indicates the new species.

The characteristics and statistics of the datasets used in this study are summarized in Table 3. ML and BI analyses of dataset of three DNA regions (ITS, *trnL-F* and *rbcL*) resulted in the same tree topologies that both indicate the new species recovering in the clade C8a of *Pilea* (PP = 1, BS = 100%) (Fig. 2).

**Table 3.** Statistics for the molecular datasets used in this study.

	Number of sequences	Aligned length (bp)	Variable characters (bp)	Parsimony information characters (bp)	Model used
ITS	142	528	419	339	-
*trnL-F*	139	677	667	38	-
*rbcL*	84	637	318	315	-
Combined	142	1,842	1,404	692	GTR+GAMMA

### ﻿Taxonomic treatment

#### ﻿ Pilea danxiaensis

Taxon classificationPlantaeRosalesUrticaceae

L.F.Fu, A.K.Monro & Y.G.Wei sp. nov.

D182AA2C-FBB8-5CDC-8DE8-84398D74B6EA

urn:lsid:ipni.org:names:77303547-1

Fig. 3

##### Type.

China. Guangdong: Danxiashan National Park, Renhua County, Shaoguan City, 25.020°N, 113.752°E (WGS84), elev. 134 m, 2 April 2022, Liao Wen-Bo, Fan Qiang and Liao Li-Juan DNPC1728 (holotype IBK; isotypes IBK, SYS).

##### Diagnosis.

Most similar to *Pileasinocrassifolia* C.J.Chen from which it can be distinguished by the longer stipule (1.3–1.5 mm versus 1 mm), petiole (2–8 mm versus 0.2–0.6 mm) and staminate peduncle (8–25 mm versus 1.5–7 mm).

##### Description.

Herbs prostrate, stem 30–200 × 1 mm, light green when fresh, drying yellowish-brown, glabrous, succulent, cystoliths fusiform, 0.2–0.4 mm long. Stipules, 1.3–1.5 × 1.7–2.1 mm, reniform, drying brown, papery, with dense cystoliths. Leaves petiolate, distichous, clustered towards the stem apex; petioles 2–8 mm long, glabrous, cystoliths densely scattered; laminae at each node equal or subequal, 3–15 × 5–20 mm, length: width ratio 0.7–0.9:1, suborbicular to broadly ovate, succulent, papery when dry; adaxial surface drying grey-green, dark green when fresh, glabrous, with cystoliths densely scattered, *ca* 0.3 mm, linear or fusiform; abaxial surface drying light green, green when fresh, glabrous, rugose when dry, 3-veined, secondary veins 3–6 pairs, borne at 45–60° to the midrib, with cystoliths sparsely scattered, *ca* 0.3 mm, linear or fusiform; apex obtuse or subretuse, base cuneate, rounded or subtruncate, margin entire and revolute. Inflorescences in upper nodes, appearing terminal, monoecious. Staminate inflorescences 10–30 mm, bearing 10–25 flowers in a capitulum or occasionally a glomerule; peduncle 8–25 × 0.5 mm, glabrous; pedicels *ca* 0.8 mm, glabrous. Staminate flowers 1 × 1 mm, green, drying light green, sepals 4, *ca* 1.8 mm; valvate, elliptic, glabrous, the subapical appendage *ca* 0.1 mm, corniculate, glabrous; stamens 4. Pistillate inflorescences 10–20 mm, bearing 20–50 flowers in a cyme or glomerule; peduncle 8–18 × ca 0.5 mm, glabrous; pedicels *ca* 0.5 mm. Pistillate flowers *ca* 0.5 mm, sepals 3, subequal, *ca* 0.3 mm, valvate, triangular-ovate, glabrous, the subapical appendage *ca* 0.1 mm. Infructescences 15–20 mm; peduncle 15–18 mm; achenes 0.68–0.72 × 0.40–0.46 mm, ovoid, spinulose-verrucose, rarely smooth.

##### Distribution and habitat.

*Pileadanxiaensis* L.F.Fu, A.K.Monro & Y.G.Wei is known from a single locality in Renhua County, Shaoguan City, Guangdong, China, where it grows in a ravine on the Danxia landform, a petrographic geomorphology formed from Cretaceous sandstones and conglomerates.

##### Phenology.

Flowering from March to May, fruiting from April to June.

##### Etymology.

The species epithet is named after the Danxia landform with which the species is associated.

##### Vernacular name.

dān xiá lěng shuǐ huā (Chinese pronunciation); 丹霞冷水花 (Chinese name).

##### Conservation status.

At present, *Pileadanxiaensis* is known from a single locality, the Danxiashan National Park. The park covers 140 km^2^ and the massif from which the type collection was made encompasses *ca* 114 km^2^ (Google Earth Pro). Within that locality, the population of *P.danxiaensis* is estimated to number between 1,000 and 5,000 individuals distributed between 10 sub-populations, of which only one has been directly observed. A remote survey of the Danxiashan National Park using Google Earth Pro, suggests that the protected area itself is well protected and we observed no active threat or continuing decline in population size. *Pileadanxiaensis* is therefore classified as Least Concern (**LC**).

##### Additional specimen examined.

China. Guangdong: Danxiashan National Geopark, Renhua County, Shaoguan City, 25.004°N, 113.655°E (WGS84), elev. 466 m, 20 April 2018, *Fan Qiang and Huang Yan-Shuang 16993* (IBK, SYS).

**Figure 3.**
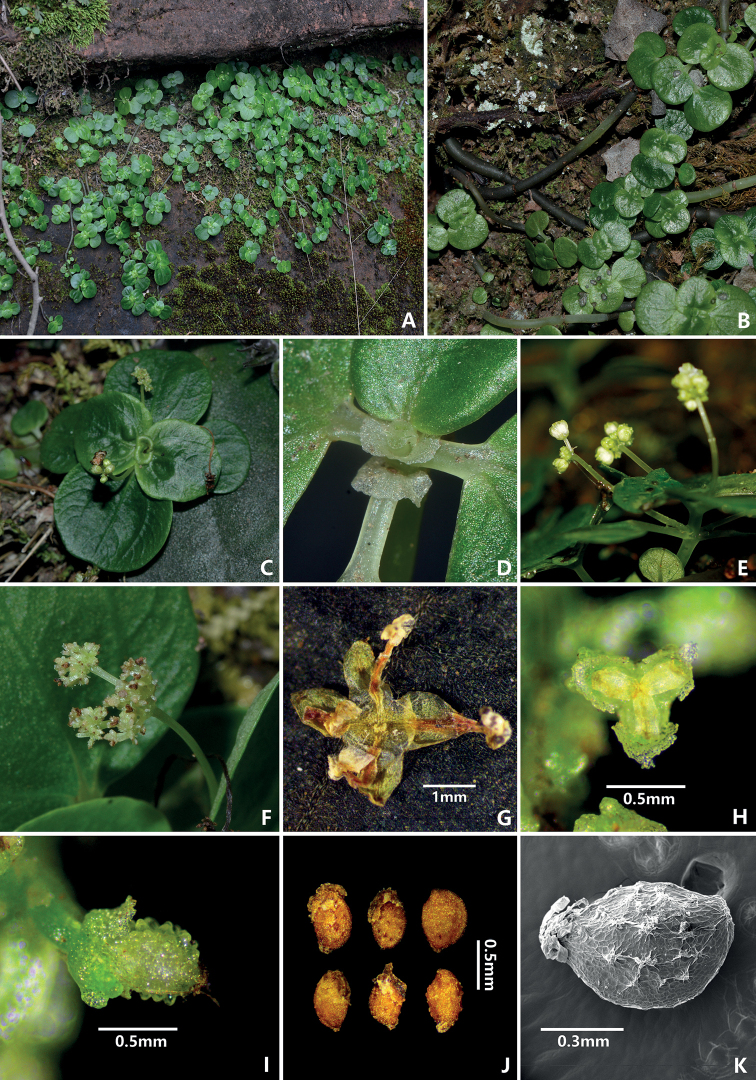
Plate of *Pileadanxiaensis***A** habitat **B** habit **C** leaves and inflorescence **D** stipules **E** staminate inflorescence **F** pistillate inflorescence **G** staminate flower **H** pistillate flower **I** achene with pistillate sepals **J** LM of achene **K**SEM of achene.

## ﻿Discussion

Fu et al. (2022) proposed a new infrageneric classification based on molecular and morphological evidence that suggested the leaf margin, stipule length, inflorescence architecture, flower sepal-number and achene ornamentation can be reliably used to place taxa into sections. Our research demonstrates that *Pileadanxiaensis* sits within clade C8a (Fig. 2) corresponding to P.sect.Pilea. Section Pilea is the most species-rich section in Pilea and has its center of species-richness in the neotropics from where several new species have been described in recent years (Monro 2006; Cabral et al. 2020; Beutelspacher and García-Martínez 2021). The morphology of *Pileadanxiaensis*, and specifically the 3-parted female flowers, 4-parted male flowers, short stipules (≤10 mm) and un-ornamented achenes, are congruent with it belonging to this section. SEM results indicate the achene length of *P.danxiaensis* to be 0.68–0.72 mm (≤ 0.8 mm), further supporting the inclusion of this species in P.sect.Pilea, and of a shift to smaller fruits as more lineages have formed (Fu et al. 2022).

Within Pileasect.Pilea, the new species is most morphologically similar to *P.sinocrassifolia* and *P.peploides* from which it is distinguished in Table 4.

**Table 4.** Diagnostic comparison of *Pileadanxiaensis*, *P.sinocrassifolia* and *P.peploides*.

Characters	*P.danxiaensis*	*P.sinocrassifolia*	*P.peploides*
Stipule shape and length	reniform, 1.3–1.5 mm	triangular, *ca* 1 mm	triangular, *ca* 0.5 mm
Petiole length	2–8 mm	0.2–0.6 mm	3–20 mm
Staminate peduncle length	8–25 mm	1.5–7 mm	2–5 mm
Pistillate tepal number	3	NA	2

## ﻿Conclusions

This study describes a new species of *Pilea* based on morphological and molecular evidence. Our results support the new infrageneric classification proposed by Fu et al. (2022). The reported plastid genome provides informative data to support further studies on the systematics, evolution, and conservation of the genus.

## Supplementary Material

XML Treatment for Pilea danxiaensis

## Supplementary materials

Supplementary material 1 Appendix S1 Data type GenBank accession numbers. Explanation note Taxa and GenBank accession numbers of DNA sequences used in this study. https://binary.pensoft.net/file/730346This dataset is made available under the Open Database License (http://opendatacommons.org/licenses/odbl/1.0/). The Open Database License (ODbL) is a license agreement intended to allow users to freely share, modify, and use this Dataset while maintaining this same freedom for others, provided that the original source and author(s) are credited.Long-Fei Fu, Chi Xiong, Alexandre Monro, Qiang Fan, Zai-Xiong Chen, Fang Wen, Zi-Bing Xin, Yi-Gang Wei, Wen-Bo Liao
