# Supplementary material for: ﻿Pileadanxiaensis (Urticaceae), a new species in the Danxia landform from Guangdong, China including a description of the entire chloroplast genome
Source: PhytoKeys. 2022 Aug 19;204:109–19. doi: 10.3897/phytokeys.204.86857 (PMC9848946; doi:10.3897/phytokeys.204.86857)
Supplement: Supplementary material 1 — Appendix S1 [file phytokeys-204-109_article-86857__-s001.docx]

Appendix I. Taxa and GenBank accession numbers of DNA sequences used in this study.

Voucher information for samples of which sequences were newly generated is given using the following format: Taxon name, molecular number, collection locality, collector and collector number (herbarium for voucher specimen), GenBank accession numbers for ITS, *trnL-trnF*, *rbcL*, respectively. Samples downloaded from NCBI only remain Taxon name, molecular number, and GenBank accession numbers as stated above. (– not available, * newly generated sequences).

*Achudemia javanica* Blume, LWQ123, MT516339, MT523094, MT523050. *Haroldiella rapaensis* J.Florence, 01731, OL310751, –, –. *Lecanthus peduncularis* (Royle) Wedd., Le1, KF137871, KF138350, KF138186. *Lecanthus petelotii* (Gagnep.) C.J.Chen var. *corniculata* C.J.Chen, Le2, KF137873, KF138352, KF138188. *Pilea alpina* Urb., 3426, DQ175543, DQ179309, –. *Pilea amplistipulata* C.J.Chen, LCN116, MT516340, MT523095, MT523051. *Pilea angulata* (Blume) Blume, LCN072, MT516341, MT523096, MT523052. *Pilea angustifolia* Killip, 26254, DQ175556, DQ179289, –. *Pilea anisophylla* Wedd., J104, MT516342, MT523097, MT523053. *Pilea aphrophila* Killip, 34825, DQ175589, DQ179323, –. *Pilea aquarum* Dunn, LCN177, MT516343, MT523098, MT523054. *Pilea aquarum* Dunn subsp. *acutidentata* C.J.Chen, LCN176, MT516344, MT523099, MT523055. *Pilea balansae* Gagnep., MT516345, MT523100, –. *Pilea basicordata* W.T.Wang, DQ175614, DQ179361, –. *Pilea benguetensis* C.B.Rob., 9696, DQ175554, DQ179337, –. *Pilea boniana* Gagnep., LCN019, MT516346, MT523101, MT523056. *Pilea boniana* Gagnep., LCN044, MT516347, MT523102, MT523057. *Pilea cadierei* Gagnep. & Guillaumin, XZB102, MT516348, MT523103, MT523058. *Pilea cavaleriei* H.Lév., P3, KF137895, KF138380, KF138214. *Pilea ciliata* Blume, 5163, DQ175538, DQ179300, –. *Pilea clementis* Britton, 690, DQ175550, DQ179310, –. *Pilea consanguinea* Wedd., 3597, DQ175539, DQ179312, –. *Pilea cordistipulata* C.J.Chen, LCN054, MT516349, MT523104, MT523059. *Pilea cordistipulata* C.J.Chen, LCN100, MT516350, MT523105, MT523060. *Pilea costata* Killip, 4015, DQ175595, DQ179290, –. *Pilea daguensis* Killip, 3844, DQ175567, DQ179332, –. *Pilea danxiaensis* L.F.Fu, A.K.Monro & Y.G.Wei, XNY205, 17556 (SYS), ON557625^*^, ON496932^*^, ON496932^*^. *Pilea dauciodora* Wedd. ex Pav., 20771, DQ175562, DQ176857, –. *Pilea digitata* A.K.Monro, 7169, DQ175559, DQ179326, –. *Pilea dolichocarpa* C.J.Chen, J101, MT516351, MT523106, MT523061. *Pilea dominguensis* Urb., 3180, DQ175541, DQ179313, –. *Pilea ecboliophylla* Donn.Sm., 1011, DQ175531, DQ179292, –. *Pilea elegantissima* C.J.Chen, P39, MH357923, MH358303, MH358124. *Pilea elliptilimba* C.J.Chen, MT516352, MT523107, –. *Pilea fairchildiana* Jestrow & Jiménez Rodr., 302A, JN252482, JN252481, –. *Pilea foliosa* Killip, 24400, DQ175571, DQ179291, –. *Pilea forgetii* N.E.Br., 3498A, DQ175585, DQ179333, –. *Pilea forsythiana* Wedd., 1196, DQ175546, DQ179311, –. *Pilea fruticosa* Hook.f., 27865, DQ175604, DQ179353, –. *Pilea gansuensis* C.J.Chen & Z.X.Peng, J330, MZ490601, MZ490605, MZ490603. *Pilea glaberrima* (Blume) Blume, 220, DQ175600, DQ179352, –. *Pilea gracilis* Hand.-Mazz., MT516353, MT523108, –. *Pilea grandifolia* Blume, 867, DQ175551, DQ179303, –. *Pilea guizhouensis* A.K.Monro, C.J.Chen & Y.G.Wei, LCN056, MT516354, MT523109, MT523062. *Pilea harrisii* Urb., 11220, DQ175537, DQ179302, –. *Pilea hexagona* C.J.Chen, LCN041, MT516355, MT523110, MT523063. *Pilea hexagona* C.J.Chen, LCN113, MT516356, MT523111, MT523064. *Pilea hilliana* Hand.-Mazz., LCN112, MT516357, MT523112, MT523065. *Pilea howelliana* Hand.-Mazz., P45, MH357926, MH358306, MH358127. *Pilea howelliana* Hand.-Mazz. var. *denticulata* C.J.Chen, LCN043, MT516358, MT523113, MT523066. *Pilea inaequalis* Wedd., 9268, DQ175552, DQ179304, –. *Pilea insolens* Wedd., LCN039, MT516359, MT523114, MT523067. *Pilea irrorata* Donn.Sm., 3919, DQ175535, DQ179294, –. *Pilea japonica* Hand.-Mazz., LCN084, MT516360, MT523115, MT523068. *Pilea krugii* Urb., 964, DQ175581, DQ179315, –. *Pilea lapestris* Chew ex A.K.Monro, 9979, DQ175598, DQ179341, –. *Pilea lindeniana* Wedd., 1449, DQ175547, DQ179314, –. *Pilea longicaulis* Hand.-Mazz., 01909, China, DQ175611, DQ179363, –. *Pilea longicaulis* Hand.-Mazz., LCN089, MT516362, MT523117, MT523069. *Pilea longicaulis* Hand.-Mazz. var. *erosa* C.J.Chen, LCN165, MT516361, MT523116, –. *Pilea longipedunculata* Chien & C.J.Chen, P5, KF137897, KF138382, KF138216. *Pilea martini* Hand.-Mazz., P6, KF137898, KF138383, KF138217. *Pilea melastomoides* (Poir.) Wedd., 12229, DQ175596, DQ179345, –. *Pilea melastomoides* (Poir.) Wedd., LCN192, MT516363, MT523118, MT523070. *Pilea melastomoides* (Poir.) Wedd., P20, KF137899, KF138384, KF138218. *Pilea mexicana* Wedd., 3512, DQ175579, DQ179278, –. *Pilea microphylla* (L.) Liebm., P101, MH357928, MH358307, MH358129. *Pilea monilifera* Hand.-Mazz., dt075, MK911055, MK911077, MK911100. *Pilea multicellularis* C.J.Chen, J113, MT516364, MT523119, MT523071. *Pilea multicellularis* C.J.Chen, LCN109, MT516365, MT523120, MT523072. *Pilea nigrescens* Urb., 543, DQ175582, DQ179301, –. *Pilea nonggangensis* Y.G.Wei, L.F.Fu & A.K.Monro, XZB101, MT516366, MT523121, MT523073. *Pilea notata* C.H.Wright, LCN175, MT516367, MT523122, MT523074. *Pilea notata* C.H.Wright, XZB103, China, Huang S.L. HSL099, MT516368, MT523123, MT523075. *Pilea nummulariifolia* (Sw.) Wedd., A.K.Monro 3989, DQ175588, DQ179316, –. *Pilea oxyodon* Wedd., P9, KF137902, KF138387, KF138221. *Pilea paniculigera* C.J.Chen, LCN166, MT516369, MT523124, MT523076. *Pilea pansamalana* Donn.Sm., 7731, DQ175533, DQ179296, –. *Pilea pellionioides* C.J.Chen, LCN052, MT516370, MT523125, MT523077. *Pilea pelonae* Urb. & Ekman, 9015, Dominican Republic, DQ175540, DQ179327, –. *Pilea peltata* Hance, LCN173, MT516371, MT523126, MT523078. *Pilea penninervis* C.J.Chen, J85, MT516372, MT523127, MT523079. *Pilea peperomiifolia* Liebm., 295, DQ175569, DQ179281, –. *Pilea peperomioides* Diels, 4182, DQ175605, DQ179350, –. *Pilea peploides* (Gaudich.) Hook. & Arn., J111, MT516373, MT523128, MT523080. *Pilea peploides* (Gaudich.) Hook. & Arn. var. *major* Wedd., P62, MH357931, –, MH358132. *Pilea pittieri* Killip, 4009, DQ175560, DQ179328, –. *Pilea plataniflora* C.H.Wright, 509, DQ175599, DQ179349, –. *Pilea plataniflora* C.H.Wright, LCN138, MT516374, MT523129, MT523081. *Pilea pleuroneura* Donn.Sm., 10110, DQ175532, DQ179297, –. *Pilea pseudonotata* C.J.Chen, LCN199, MT516375, MT523130, MT523082. *Pilea pubescens* Liebm., 2663, DQ175558, DQ179325, –. *Pilea pumila* A.Gray, LCN151, MT516376, MT523131, MT523083. *Pilea pumila* A.Gray, P64, MH357932, MH358309, MH358133. *Pilea racemiformis* C.J.Chen, J91, MT516377, MT523132, MT523084. *Pilea racemosa* (Royle) Tuyama, 2686, DQ175602, DQ179347, –. *Pilea receptacularis* C.J.Chen, 32530, DQ175612, DQ179362, –. *Pilea rivularis* Wedd., 935, DQ175606, DQ179358, –. *Pilea rufa* Wedd., 5147, DQ175578, DQ179299, –. *Pilea semisessilis* Hand.-Mazz., LCN107, MT516378, MT523133, MT523085. *Pilea sinocrassifolia* C.J.Chen, J264, Guangxi, China, Fu L.F. and Xin Z.B. FLF190801-02 (IBK), ON557627^*^, ON496934^*^, ON496934^*^. *Pilea sinocrassifolia* C.J.Chen, XNY206, Guangdong, China, Fan Q. 17792 (SYS), ON557626^*^, ON496933^*^, ON496933^*^. *Pilea sinofasciata* C.J.Chen, P26, KF137905, KF138389, KF138224. *Pilea* sp., LCN059, MT516379, MT523134, MT523086. *Pilea* sp., DQ175601, DQ179344, –. *Pilea spathulifolia* Groult, 8148, DQ175570, DQ179282, –. *Pilea spicata* C.J.Chen & A.K.Monro, LCN105, MT516380, MT523135, MT523087. *Pilea subcoriacea* (Hand.-Mazz.) C.J.Chen, LCN060, MT516381, MT523136, MT523088. *Pilea succulent* Wedd., 29043, DQ175565, DQ179280, –. *Pilea swinglei* Merr., P66, MH357933, –, MH358134. *Pilea ternifolia* Wedd., 390, DQ175597, DQ179346, –. *Pilea tetraphylla* Blume, J287, MZ490600, MZ490604, MZ490602. *Pilea thymifolia* Ridl., 3921, DQ175568, DQ179283, –. *Pilea tridentata* Killip, 3088, DQ175536, DQ179293, –. *Pilea tripartita* A.K.Monro, 4181, DQ175617, DQ176859, –. *Pilea tsiangiana* F.P.Metcalf, LCN049, MT516382, MT523137, MT523089. *Pilea umbrosa* Wedd., J97, MT516383, MT523138, MT523090. *Pilea unciformis* C.J.Chen, LCN156, MT516384, MT523139, MT523091. *Pilea villicaulis* Hand.-Mazz., LCN038, MT516385,MT523140,MT523092. *Pilea virgate* Wedd., 15152, DQ175548, DQ179329, –. *Pilea vulcanica* Liebm., 3511, DQ175563, DQ179284, –. *Pilea weddellii* Fawc.& Rendle, 4948, DQ175545, DQ179308, –. *Pilea weimingii* Huan C.Wang, LCN047, MT516386, MT523141, MT523093. *Boehmeria japonica* (L.f.) Miq., B47, KF137808, KF138279, KF138116. *Cannabis sativa* L., Z1773, MH357863, –, MH358052. *Cecropia ficifolia* Warb. ex Snethl., 23606, KF137825, KF138296, KF138133. *Droguetia iners* (Forssk.) Schweinf., Dr1, KF137844, KF138318, KF138154. *Elatostema cyrtandrifolium* (Zoll. & Moritzi) Miq., E3, KF137848, KF138322, KF138158. *Elatostema parvum* (Blume) Blume ex Miq., E7, KF137852, KF138326, KF138162. *Fatoua villosa* Nakai, F1, KF137858, KF138331, KF138168. *Humulus lupulus* L., D3848, MH357893, –, MH358086. *Morus alba* L., HM747164, HM747180, L01933. *Myriocarpa cordifolia* Liebm., C2A, KF137877, KF138357, KF138193. *Nanocnide japonica* Blume, N1, KF137879, KF138359, KF138194. *Pellionia radicans* Wedd., Pe3, KF137891, KF138375, KF138210. *Pellionia repens* (Lour.) Merr., Pe4, KF137892, KF138376, KF138211. *Pellionia tsoongii* Merr., Pe5, KF137893, KF138377, KF138212. *Poikilospermum lanceolatum* (Trécul) Merr., Pi1, China, KF137912, KF138396, KF138231. *Procris crenata* C.B.Rob., Pr1, KF137922, KF138407, KF138242. *Rousselia humilis* (Sw.) Urb., Dominica, KM586474, KM586646, KM586560. *Sorocea affinis* Hemsl., HM747179, HM747195, GQ981880. *Trophis racemosa* Urb., HM747178,HM747194, GQ981908.
